# Would You Consider Becoming a Rheumatologist? A Survey Regarding the Attractiveness of Rheumatology as a Career Among Moroccan Medical Students

**DOI:** 10.31138/mjr.20230727.wyc

**Published:** 2023-07-27

**Authors:** Fatima Zahrae Taik, Anass Adnine, Hatime Tbatou, Amine Amar, Fatima Ezzahra Abourazzak

**Affiliations:** 1 Department of Rheumatology, Tangier-Tetouan-El Hoceima University Hospital, Tangier, Morocco,; 2 Faculty of Medicine and Pharmacy of Tangier, Abdelmalek Essaadi University, Tetouan, Morocco,; 3 Applied Mathematics and Data Science, School of Science and Engineering, Al Akhawayn University, Morocco

**Keywords:** rheumatology, medical students, specialty choice

## Abstract

**Objective:**

To assess the attractiveness of a career in rheumatology among Moroccan medical students and to study factors that motivate or demotivate them to choose rheumatology as a future career.

**Methods:**

An electronic survey was distributed among students in medical training, interns, and graduates from the Faculty of Medicine at the University Hospital of Tangier. The questionnaire evaluated the level of clinical exposure to rheumatology, the interest in rheumatology as a specialty, and the motivation or demotivation for choosing or not choosing rheumatology as a career.

**Results:**

318 students responded to the survey. Of these, 57.5% reported that they had already completed a training period in a rheumatology department. Of Moroccan students, 35.6% would consider specialising in rheumatology and 8.5% of these stated that rheumatology was their first specialty choice. The uni- and multi-variate analysis determined that clinical exposure to rheumatology (OR=2.39 IC95% [1.46–3.91]) and female gender (OR=1.95 IC95% [1.2–3.2]) were the main statistically significant factors for the prediction of the choice of rheumatology. Intern status was statistically associated with not choosing rheumatology (OR=0.085 IC95% [0.03–0.24]. The main motivation factors for Moroccan medical students to choose rheumatology were the diversity of musculoskeletal diseases (54.5%) and the good balance work/life (46.6%). The main reasons for not choosing rheumatology were the limited therapeutic aspects of the discipline (30.3%) and an interest in surgical specialties (29.3%).

**Conclusion:**

Rheumatology fascinates Moroccan medical students by the diversity of its pathologies and the good balance work/life. Greater clinical exposure to rheumatology is the strongest predictor of medical students choosing rheumatology as a future career.

## INTRODUCTION

Rheumatology is a young (born in middle of the 20th century!^1^) but unique and challenging specialty that has developed significantly in recent decades, notably with the use of musculoskeletal ultrasound in routine clinical practice^2,3^ and the introduction of biotherapy and targeted therapy.^4^ However, despite these positive advances, publications from some developed countries (Canada, USA, UK, Belgium) have shown that medical students are not attracted to the specialty of rheumatology as a career.^5–10^ No studies have yet been conducted in emerging countries such as in Morocco to investigate the motivations and demotivations of medical students for the choice of rheumatology as a specialty. Indeed, rheumatology in emerging countries still faces many challenges.^11^ The deficient health systems in most of these countries, tends to give priority to other more “vital” medical specialties and thus, if we analyse, for example, the total number of rheumatologists in Morocco, we found that only 370 rheumatologists exist, including 96 undergoing specialisation; ie, less than 1 rheumatologist per 100000 people. Furthermore, medical education issues, such as the disproportional limited number of academic centres (especially academic rheumatology centres), comparatively to the increasing numbers of medical students, linked essentially with the urgent need to train practitioners, would probably be a major factor which limits clinical exposure to rheumatology. In this context, our objective was to assess the attractiveness of rheumatology among Moroccan medical students, interns, and graduates and to identify, in addition, the main factors that motivate or demotivate them, when making decisions about the rheumatology as a future career.

## METHODS

### Study Design

An electronic questionnaire-based survey was conducted among three categories of medical students in the Faculty of Medicine and Pharmacy of Tangier between January and April 2021. Permission for the study was obtained from the Dean of the Faculty of Medicine. The study protocol was exempted from the review of the University Hospital Ethics Committee of Tangier since it was an online survey of medical students. Ethics waiver protocol number: EX01SP/22. The study was performed in accordance with the ethical standards as laid down in the 1964 Declaration of Helsinki. A response to the questionnaire was considered as consent for participation. Anonymity and confidentiality of the responses were maintained (we did not collect participants’ names or phone numbers and the data collection tool used was set to not collect participants’ e-mail).

### Population

Our survey targeted three categories of students, namely 1) 297 second cycle students from the Faculty of Medicine of Tangier (3^rd^, 4^th^, and 5^th^ year), 2) 100 medical interns and 3) 180 medical graduates who had passed the residency competition in the December 2021 session (before choosing their specialty).

The duration of medical studies in Morocco varies between 8 years (general medicine) to 13 years (specialty), and it is organised as follows: a first cycle of pre-clinical sciences, a second cycle for clinical studies and hospital training, and then two years of full-time training, at the end of which students complete their clinical exams and defend their theses to qualify as a Doctor of Medicine. There are two ways to be admitted to a medical specialty, namely via an internship or via a residency competition. Students who have successfully completed their fifth year and have validated all the training periods can pass the internship competition. Interns complete two years of internship in university hospitals, at the end of which they are appointed as residents and have a free choice of specialty. Those who do not follow the internship pathway should defend their theses and pass the residency competition to be nominated as residents through the competition. The choice of specialty is awarded according to merit.

### Questionnaire

The authors (two professors of rheumatology, one intern, and one resident in rheumatology) and two students developed the first version of the study questionnaire based on the results of previous studies.^5–10^ Then we conducted a cognitive interview with five students and six residents in rheumatology, about motivations and demotivations for choosing rheumatology as a specialty. Participants were asked to provide feedback on each question (acceptability and clarity) and to indicate if there were any questions that could be added or removed. Some changes were made thereafter. Then the questionnaire was reviewed by a committee (4 professors of rheumatology) who approved the content of the questionnaire. Finally, we conducted a pilot study with a group of 30 students to evaluate the acceptability and the usability of the questionnaire and to identify possible others’ misunderstandings and misinterpretations. All participants stated that the questionnaire was understandable without any ambiguity. Test and retest reliability for single item correlations were strong or very strong and statistically significant (p < 0.001). Construct validity was evaluated by performing a logistic regression that showed the results we expected and that have already been found in the literature.

The final version of the questionnaire consisted of 26 questions distributed in four parts, namely 1) demographic information, 2) information regarding the degree of clinical exposure to rheumatology, 3) attitudes and beliefs toward rheumatology as a specialty (participants were asked about the importance of rheumatology for their career and whether they consider rheumatology as a future career) and 4) the main motivations and demotivations for the choice of rheumatology as a specialty. The complete questionnaire is detailed in **Appendix 1**.

Three minutes was sufficient time to complete the questionnaire.

### Data collection

We used the online survey tool “Google Forms” for data collection. The link was sent to student representatives and the presidents of the intern and resident associations with a cover letter that explained the purpose and objectives of our survey. We asked them to distribute the link to all the students, interns, and those recently admitted to the residency competition via either e-mail or WhatsApp. The data collection was done between 01/12/2021 and 04/20/2021.

### Statistical analysis

A descriptive analysis was conducted for the entire population, using headcount and percentage for qualitative variables and median and standard deviation for quantitative variables, if they were normally distributed; otherwise, they were expressed in medians and quartiles. Chi-squared tests were used to assess the differences between the groups (based on gender and exposure to rheumatology). Univariate and multivariate logistic regression analysis were performed to study associations between demographic variables, year of training, exposure to rheumatology, and choosing rheumatology as a future career. Data were analysed using SPSS_21.0, and the test results were considered significant if p < 0.05.

## RESULTS

### Demographic characteristics

The response rate was 57.1%. The mean age was 24.23±3.06. A slight predominance of females was noted (58.8%). In our sample, 50.6% of respondents were second cycle students, 28% were graduates, and 21.4% were interns. The demographic characteristics are presented in **Table 1**.

**Table 1.** Demographic characteristics of participants.

	**N= 318**

**Age^a^**	24.23±3.06

**Female gender (%)^b^**	187 (58.8)

**Status (%)^b^**	
- **2nd cycle students**	161 (50.6)
○ **3^rd^ year**	44 (13.8)
○ **4^th^ year**	53 (16.6)
○ **5^th^ year**	64 (20.1)
- **Interns**	68 (21.4)
- **Graduates**	89 (28)

**Prior training in a rheumatology department (%)^b^**	183 (57.5)

**Duration of rheumatology training period (weeks)^a^**	5±1.56

Values are given as mean±standard deviation (^a^) and frequency (^b^).

### Clinical exposure to rheumatology

More than half of the participants (57.5%) reported that they had already completed a training period in a rheumatology department. The mean duration of the training period was 5±1.56 weeks (**Table 1**).

### Choosing rheumatology as a future specialty

One hundred and ten (34.6%) of the respondents stated that they were considering rheumatology as a specialty. In total, 41.6% of the second cycle students, 5.9% of the interns, and 43.8% of the medical graduates stated that rheumatology was among their specialty choices, while 11.2% of the students, 5.9% of the interns, and 7.9% of the graduates stated that rheumatology was their first specialty choice.

From uni- and multi-variate analysis, clinical exposure to rheumatology (OR=2.39 IC95% [1.46–3.91]; p=0.001) and female gender (OR=1.95 IC95% [1.2–3.2]; p=0. 001) were retained as the main statistically significant factors for predicting the possibility of considering rheumatology as a career. Intern status was statistically associated with not choosing rheumatology (OR=0.085 IC95% [0.03–0.24]; p<0.001) (**Table 2**).

**Table 2.** Univariate and multivariate analysis for choosing rheumatology as a career.

	**Univariate analysis**		**Multivariate analysis**	
	OR (IC 95%)	*p*	OR (IC 95%)	*p*
**Age**	1.03 (0.96–1.1)	0.35	-	-
**Gender**	1.95 (1.2–3.2)	**0.007**	1.72 (1.02–2.89)	**0.04**
**Intern status**	0.085(0.03–0.24)	**<0.001**	0.1 (0.03–0.29)	**<0.001**
**Prior training in a rheumatology department**	2.39 (1.46–3.91)	**0.001**	2.08 (1.23–3.51)	**0.006**
**Duration of training period**	1.21 (0.73–2.01)	0.44	-	-

OR: odds ratios; CI: confidence interval.

### Motivating factors for choosing or not choosing rheumatology as a specialty

The main motivation of Moroccan medical students to choose rheumatology was that it is a “fascinating specialty with diversity of musculoskeletal diseases” (according to 54.5% of students who will consider becoming a rheumatologist). The second advantage of rheumatology was that it allows for a good balance between workload and family time (46.4% of respondents who plan to choose rheumatology). Third, the importance of the clinical examination in the diagnostic process seemed to motivate 42.7% of the students who are considering choosing rheumatology.

The main barrier to choosing rheumatology as specialty was the “limited therapeutic options,” according to 30.3% of students who would not choose rheumatology. The second reason for not choosing rheumatology was an interest in surgical specialties, according to 29.3% of the respondents who would not choose rheumatology. Other frequently reported reasons are presented in **Table 3**.

**Table 3.** Main motivations and demotivations for choosing rheumatology according to Moroccan medical students.

	**N=318**
**Motivations**	N=110
- Fascinating specialty with diversity of musculoskeletal diseases	60 (54.5 %)
- Good balance between workload and family time	51 (46.4 %)
- Clinical aspects of the specialty; anamnesis and clinical examination are extremely important in the diagnostic process	47 (42.7%)
- Interventional aspect of the discipline: joint puncture, synovial biopsy, infiltration, arthrodistension, and more	36 (32.8%)
- Development of exploration techniques for the musculoskeletal system: osteoarticular ultrasound, bone exploration, new imaging techniques, and more	25 (22.7%)
- The development of new therapies (targeted therapies)	20 (18.2%)
- Financial compensation	19 (17.3%)
**Demotivations**	N=208
- Limited therapeutic options	63 (30.3 %)
- I am interested in surgical specialties	61 (29.3 %)
- Features of the specialty: long and complicated rheumatological examination, need for a good knowledge of anatomy, and more	49 (23,6 %)
- Boring specialty	46 (22.7%)
- Patient profile (geriatric population, and more)	41 (19.7%)
- Too many chronic diseases	36(17.3%)
- Financial compensation	30 (14.4%)

## DISCUSSION

With this survey, we wanted to study the attractiveness of rheumatology to Moroccan medical students, the factors associated with the attractiveness or lack thereof, and students’ motivations and demotivations for choosing or not choosing rheumatology as a career. Our results showed that 35.6% of Moroccan students would consider becoming a rheumatologist. In addition, 8.5% of the respondents stated that rheumatology was their first specialty choice. We found that the main factors associated with the choice of rheumatology were clinical exposure to rheumatology and female gender, whereas the internship pathway was associated with not choosing rheumatology. The most reported reasons that motivate Moroccan students to choose rheumatology were the diversity of pathologies, the clinical aspect of the specialty, and the good balance between workload and family time that it allows. The main reasons for not choosing rheumatology were an interest in surgical specialties and the limited therapeutic aspects of the discipline.

The attractiveness of rheumatology to medical students has not yet been well studied in the literature. In a study published in 2017, Wettoek et al. reported that only 10% of Belgian medical students and 16% of internal medicine trainees considered choosing rheumatology as a specialty,^7^ while Watson and Gaffney found that 67% of doctors at foundation year 1, foundation year 2, core medical training, and basic specialty training levels consider/or have considered choosing the specialty as a career.^8^ In Canada, Katz et al. studied the attractiveness of rheumatology in a different way by calculating the percentage of internal medicine residents who chose to subspecialise in rheumatology after completing an internship in a rheumatology department. They report that, among internal medicine trainees at 13 English-speaking Canadian internal medicine accredited programs, only 3.5% entered a rheumatology training program and 78% of theme chose rheumatology as a subspecialty career.^9^ In the same way, West et al. have recently reported similar results, with only 3.2% of American-trained residents choosing a career in rheumatology.^10^

In a Spanish study published in 2018, Andréu et al. evaluated the progression of the attractiveness of rheumatology among medical specialty training candidates between 1983 and 2014 by examining the evolution of the highest, median, and lowest rank of candidates choosing rheumatology training positions in every qualifying exam call from 1983 to 2014. The authors found that, for the median of the election of rheumatology, the range increased from 244^th^ in 1983 to 3394^th^ in 2008, with an improving trend from 2009 to 2014.^12^

Prior clinical exposure has been identified as the most important factor in the selection of a specialty in several studies. Thapper and Roussou have showed that exposure to rheumatology was the most important factor in the choice of a rheumatology rotation during foundation years.^6^ Similarly, Wettoek et al. showed that early exposure was the strongest predictive factor that may attract more students to consider rheumatology as a career.^7^ The clinical training also improves the perception of rheumatology among students, as shown by Courties et al. in a recent study.^13^ At our university, our students have adequate exposure to rheumatology through interactive courses, seminars, and hospital training, which may explain their interest in rheumatology.

Female gender was also found to be a factor associated with the choice of rheumatology in our study. Indeed, in Morocco, more than 90% of Moroccan rheumatologists are women. This result was not reported by Wettoek et al.^7^ The last factor influencing the choice of rheumatology in our study was internship. Indeed, our study showed that being an intern was predictive of not choosing rheumatology as specialty (only 5.9% of interns considered the possibility of becoming a rheumatologist). This can be explained by the fact that, during the two years of internship, interns are overexposed to other specialties (such as cardiology, surgery, pediatrics, and more) while only a few do an internship rotation in rheumatology (7.4% in our sample). This issue has already been reported in a Canadian study that showed that intensive and delayed exposure (first year of internal medicine training) was one of the important factors that attract internal medicine trainees to rheumatology.^9^

Regarding the motivations for the choice of rheumatology, our specialty seems to attract Moroccan students because of its diversity of pathologies and clinical aspects in addition to the positive work-life balance. The diversity of pathologies and the balance between family and professional life, as well as therapeutic advances in rheumatology, have also been reported as the main reasons that motivate Belgian trainees in internal medicine. Watson and Gafney^8^ found that, during year 3 of specialty training, doctors consider choosing rheumatology because of an interest in the specialty and the favourable working hours. The same motivations were reported by rheumatology fellows in a study conducted by Kolasinski et al.^14^ Quality of life was also the primary motivator for Canadian residents to choose rheumatology.^15^

Regarding the motivations for not choosing rheumatology, “limited therapeutic aspects” were the main reason evoked by Moroccan students who were not interested in a rheumatology career. This negative perception was also mentioned in a study by Dunkly et al., in which they found that the main reason for not choosing rheumatology was the monotonous character of the specialty.^5^ This again refers to the perception of rheumatology among medical students, which was examined in a recent study.^13^ The authors concluded that medical students have little knowledge of rheumatology and have a negative perception of it in the absence of a rheumatology rotation. Other studies mentioned a lack of interest in musculoskeletal pathologies^7^ or an interest in another specialty^15^ as demotivations.

Economic concerns, which was mentioned by 17.3% of the students who were considering a career as a rheumatologist and 14.4% of those who were not, do not seem to be a consideration in the choice of rheumatology. Economic considerations are one of the factors that influence the choice of specialty.^16^ Indeed, high-income specialties seem to be more attractive to medical students and trainees.^17^

Our study has some limitations. The main limitation is related to the methodology. Although the questionnaire was reviewed by a faculty committee and previously evaluated in a pilot study, we did not validate the questionnaire before using it in this study. The second limitation is related to our study population. We only included students from the medical school in Tangier and, therefore, cannot extrapolate the results nationally.

In conclusion, our study showed that rheumatology fascinates Moroccan medical students by its clinical richness and the diversity of its pathologies, and also because of the good balance between workload and family time that it allows. According to the students, the main obstacles to choosing rheumatology as a specialty are an interest in a surgical specialty or the perceived limited therapeutic aspects of the discipline. Efforts should be made to improve the attractiveness of the specialty by increasing clinical exposure of medical students and interns to rheumatology, as this is the strongest predictor of choosing rheumatology as a future career.

## Appendix 1

The first part of the questionnaire related to the students’ demographic information, namely, age, gender, statute, and year of training.

The second part of the questionnaire evaluated the level of clinical exposure to rheumatology.

Have you ever completed a training period in a rheumatology department?

**Table:**

Yes	No

If yes, what was the duration of this training period? (in weeks)

For interns, have you ever done a rotation in a rheumatology department during your internship?

**Table:**

Yes	No

If not, will you consider rheumatology as a medical rotation?

**Table:**

Yes	No

The third part was related to students’ attitudes and beliefs towards rheumatology as a specialty.

Do you consider a training period in a rheumatology department to be important/useful for your career?

**Table:**

Yes	No

If yes, indicate on a scale of 0 (not important/not useful) to 10 (very important/very useful) the degree of importance.

If not, please explain (open answer)

Will you consider choosing rheumatology as a specialty?

**Table:**

Yes	No

The last questions were about students’ motivations or demotivations for choosing or not choosing rheumatology as a specialty:

Will you consider choosing rheumatology as a specialty?

**Table:**

Yes	No

*If yes, why? (multiple choice answers)*

- Fascinating specialty with diversity of musculoskeletal diseases
- Clinical aspects of the specialty; anamnesis and clinical examination are extremely important in the diagnostic process
- Development of exploration techniques for the musculoskeletal system: osteoarticular ultrasound, bone exploration, new imaging techniques and more.
- Interventional aspect of the discipline: joint puncture, synovial biopsy, infiltration, arthrodistension, and more
- The development of new therapies (targeted therapies)
- Good balance between workload and family time
- Financial compensation
- Others (open answer)

*If no, why (multiple choice answers)?*

- Boring specialty
- Features of the specialty: long and complicated rheumatological examination, need for a good knowledge of anatomy and more
- Highly specialised
- Too many chronic diseases
- Limited therapeutic options
- Patient profile (geriatric population, and more)
- Financial compensation
- I am interested in surgical specialties
- Others (open answer)
